# Stability of Monkeypox Virus on Commonly Contacted Surfaces in Clinical Settings

**DOI:** 10.1093/ofid/ofaf225

**Published:** 2025-04-16

**Authors:** Andra Banete, Jacklyn R Hurst, Winfield Yim, Emily Chien, Kuganya Nirmalarajah, Robert A Kozak, Samira Mubareka

**Affiliations:** Biological Sciences, Sunnybrook Research Institute, Sunnybrook Hospital, Toronto, Ontario, Canada; Biological Sciences, Sunnybrook Research Institute, Sunnybrook Hospital, Toronto, Ontario, Canada; Biological Sciences, Sunnybrook Research Institute, Sunnybrook Hospital, Toronto, Ontario, Canada; Biological Sciences, Sunnybrook Research Institute, Sunnybrook Hospital, Toronto, Ontario, Canada; Biological Sciences, Sunnybrook Research Institute, Sunnybrook Hospital, Toronto, Ontario, Canada; Biological Sciences, Sunnybrook Research Institute, Sunnybrook Hospital, Toronto, Ontario, Canada; Biological Sciences, Sunnybrook Research Institute, Sunnybrook Hospital, Toronto, Ontario, Canada

**Keywords:** mpox, mpox virus, stability, surface, transmission

## Abstract

Monkeypox virus (MPXV), the etiologic agent of mpox, is an enveloped DNA virus that may persist on various surfaces, contributing to fomite-mediated transmission. The ongoing global outbreak of mpox has highlighted an urgent need to understand the environmental stability of MPXV. This study investigates the stability of MPXV on surfaces encountered in clinical settings. The persistence of viable MPXV and viral DNA was evaluated using porous (gauze, cotton, and scrubs), and nonporous (stainless steel, polypropylene plastic, intravenous tubing, N95 masks, and nitrile gloves) materials. Surfaces were inoculated with 10^5^, 10^6^, and 10^7^ TCID_50_ (50% Tissue Culture Infectious Dose) MPXV and incubated at room temperature (22 °C) and 4 °C for up to 21 days to determine the effect of temperature and inoculum titre on virus viability. We show that MPXV stability is influenced by both surface type and temperature, with nonporous surfaces and lower temperatures supporting longer virus viability. Infectious MPXV was detected for up to 21 days on intravenous tubing and nitrile gloves at 4 °C, whereas porous materials like cotton showed rapid loss of infectivity, especially at room temperature. Notably, we found that viral DNA did not correlate with the presence of infectious virus, suggesting that molecular assays may overestimate fomite-mediated transmission risks. Our findings provide novel insights into MPXV persistence in clinical environments, extending prior knowledge by systematically quantifying virus viability across multiple surface types and temperature conditions. These findings underscore the importance of stringent decontamination protocols in clinical settings and highlight the need for comprehensive methods for risk assessment to evaluate the potential for MPXV transmission from contaminated surfaces.

## Teaser

Our study found that Monkeypox virus (MPXV) remains viable longer on non-porous surfaces and at lower temperatures, with infectious virus detectable up to 21 days on materials including nitrile gloves at 4 °C. The research highlights that viral DNA detection does not always indicate the presence of infectious virus, underscoring the need for accurate detection methods and effective waste management in healthcare and other settings where these materials are used.

## BACKGROUND

The disease mpox (formerly monkeypox) caused by the monkeypox virus (MPXV) has spread globally since 2022, infecting more than 127 960 people as of January 2025 (https://worldhealthorg.shinyapps.io/mpx_global), and representing the largest known human outbreak of mpox to date. MPXV is an enveloped, double-stranded DNA orthopoxvirus of the *Poxviridae* family. Small rodents and other mammals are thought to maintain MPXV in endemic areas in West and Central Africa, although the precise animal reservoir(s) remains unknown [1]. Viral transmission primarily occurs by direct contact with infectious active lesions, scabs, mucous membranes, or bodily fluids of infected humans or animals, consumption of infected meat, and hypothetically exposure by the respiratory route during prolonged close contact [2, 3].

Generally, poxviruses have high environmental stability, which has been shown to strongly depend on ambient temperature and humidity [4, 5]. Studies have shown that MPXV viability is greater at lower temperatures, with virus stability lasting more than 49 days at 4 °C, and up to 42 days at 37 °C or room temperature (RT) [6]. Due to the high environmental stability of poxviruses, it is essential to characterize the persistence on materials that may act as fomites to minimize transmission. Like other poxviruses, such as variola and vaccinia virus (VACV), MPXV is stable on stainless steel and glass for up to 10 days at RT, and up to 30 days at 4°C in laboratory studies [6–9]. Additionally, low amounts of viable MPXV have been detected on household surfaces up to 15 days after initial discovery, with surfaces such as glass, steel, and plastic showing higher detection of the virus over longer periods [10]. However, limited data are available on the persistence and viability of MPXV on materials that may act as fomites in clinical settings, and the risk they pose for transmission to healthcare workers. Healthcare workers in endemic areas are known to have a higher risk of contracting MPXV infection than the general public [11]. Although the risk is considered low [12], cases of healthcare workers infected through contact with fomites contaminated with infectious MPXV have been reported [13, 14].

Here, we evaluate the stability of infectious MPXV and correlation with nucleic acid persistence on commonly contacted porous and non-porous surfaces found in clinical settings under different environmental conditions. Our findings describe surface- and temperature-dependent viability of MPXV, which may provide insights for the guidance on specimen handling and transportation in diagnostic laboratories, Infection Prevention and Control measures, and waste management in healthcare settings.

## METHODS

### Cell Culture, Virus Propagation, and Titration

African green monkey kidney VeroE6 cells (ATCC # CRL1586) were cultured in Dulbecco Modified Eagle Medium (DMEM) (Wisent Bioproducts) supplemented with 10% fetal bovine serum (FBS) (Wisent Bioproducts), 1x penicillin and streptomycin, and L-glutamine at 37 °C, 5% CO_2_. MPXV was isolated from an inguinal lesion swab sample and passaged on Vero E6 cells as previously described [15]. VeroE6 cells were seeded onto 10 × 10-cm cell culture dishes in DMEM + 10% FBS to 90%–100% confluency after 24 hours. After 24 hours, cell culture media was removed, and the virus sample was brought to 2 mL in DMEM + 10% FBS and overlaid onto cells. Culture dishes were then transferred to a 37 °C incubator with 5% CO_2_ for 1 hour with gentle shaking every 10 minutes. After 1 hour incubation, 8 mL of DMEM + 10% FBS were added to the infected cells and returned to the incubator. Cells were monitored daily for cytopathic effect (CPE) and supernatant was collected after 72 hours when CPE was greater than 50%. The infected cells and supernatant were centrifuged at 2000 rpm for 5 minutes at 4 °C. The pellet was discarded, and virus-containing supernatant was saved and stored at −80 °C. The final MPXV stock was amplified to passage 3 on VeroE6 cells in a confluent T175 flask. After 5 days, infected cells and supernatant were harvested and underwent 3 freeze-thaw cycles. The virus stock was centrifuged as described previously, aliquoted, and stored at −80 °C.

Virus titers were determined by the TCID_50_, an endpoint dilution assay to determine the viral dose in which 50% of the wells infected display cytopathic effect (CPE). Briefly, virus stock was 10-fold serially diluted in DMEM supplemented with 1× L-glutamine, antibiotics as described previously, and 1% FBS. Dilutions were then added to confluent VeroE6 cells in triplicate in 96-well flat bottom plates, and CPE was recorded at 6 days post-infection (dpi). The improved Karber method was used to calculate TCID_50_/mL [16].

All infectious work was performed under biosafety level 3 conditions at the Toronto High Containment Laboratory, Temerty Faculty of Medicine, University of Toronto, in accordance with the Canadian Biosafety Standard and the Human Pathogens and Toxins Act.

### Whole Genome Sequencing

The viral DNA was sequenced by whole genome sequencing as described later at Sunnybrook Health Science Center (Toronto, Ontario) to ensure the resulting viral stock was representative of the circulating clade IIb lineage B.1 variant, using previously published protocols [17]. Briefly, nucleic acids were extracted from the collected lesion swab sample using the QIAamp Viral RNA Mini Kit (Qiagen, Hilden, Germany) according to the manufacturer's protocol for purification of viral nucleic acids from plasma, serum, and cell-free body fluids. The eluate underwent cDNA conversion using Q5 Hot Start High-Fidelity DNA polymerase (New England Biolabs) and was subsequently used in 2 multiplex polymerase chain reactions (PCRs) to generate ∼5000 bp amplicons. PCR conditions were as follows: 98 °C for 2 minutes, 40 cycles of 98 °C for 10 seconds, and 65 °C for 5 minutes (with an additional 10 seconds per cycle), followed by 72 °C for 5 minutes and 4 °C for 5 minutes. PCR products were pooled and a 0.6× ratio clean-up was performed with AmpureXP Beads (Beckman Coulter). The quantity of cleaned amplicons was determined with the Qubit fluorometer using the 1X dsDNA HS Assay Kit (Thermo Fisher Scientific). Cleaned amplicons were used for sequencing library preparation with the Illumina DNA prep kit (Illumina) per manufacturer's instructions. Sequencing libraries were loaded on Miniseq with a 300-cycle reagent kit (Illumina) for paired-end sequencing.

### Bioinformatics

To confirm the lineage of the isolated MPXV from the lesion swab sample, paired-end sequencing reads were subjected to the following bioinformatics pipeline. Initially, sequencing adapters and low-quality bases were removed using Trim Galore v0.6.10 [18]. Host DNA contamination was mitigated by mapping reads to the human reference genome using the Burrows-Wheeler Aligner v0.7.17-r1188 to remove any host reads. Subsequently, viral reads were mapped to the MPXV reference genome ON694331.1 using Minimap2 v2.26-r1175. SAMtools v1.17 was employed for sorting and indexing the resulting BAM files. To refine the alignment, primer sequences were trimmed using iVar v1.4.2 [19]. Consensus generation was performed using BCFtools v1.17, followed by quality trimming and masking with Seqtk v1.4-r122 (https://github.com/lh3/seqtk).

### Material Preparation

This study evaluated the surface stability of MPXV on a total of 8 surface conditions. Porous surfaces included cotton gauze (Medline), scrubs cotton material, and paper bedding (Pro Advantage). Nonporous material included stainless steel (20-mm diameter, Systems for Research Corp), intravenous tubing (source from ICU Medical Inc), nitrile gloves (Ultident), polypropylene plastic (12-well plate, Sarstedt), and N95 masks (3M VFlex 1804). Gauze, scrubs, paper bedding, and N95 masks were cut into circular pieces with a 20-mm diameter leather hollow punch (Harfington). All surfaces except for stainless steel discs were gamma-irradiated with 5 millirads to inactivate possible biological contaminants, whereas stainless steel discs were placed in 70% ethanol then dried. Pieces were placed in a 12-well plate for inoculation with MPXV dilutions in triplicate.

### Virus Inoculation and Recovery

Inoculates of 50 µL containing 10^5^, 10^6^, or 10^7^ TCID_50_ were evenly applied to surfaces in triplicate and incubated at RT (22 °C) and 4 °C. Virus was recovered at different time points (0, 1, 3, 5, 7, 10, 14, and 21 days) by eluting each piece with 1 mL of DMEM with 1% FBS and 1x penicillin/streptomycin, and glutamate. Porous surfaces were transferred to the 2-mL screw cap collection tube, and pulse vortexed and centrifuged at 2400 rpm for 5 minutes. Nonporous surfaces were washed 10× with 1 ml DMEM with 1% FBS and 1× penicillin/streptomycin, and glutamate to elute the virus. The day 0 time point was collected immediately after addition of viral inoculum. Viral titers were quantified by TCID_50_ as described previously to determine infectious virus.

### Quantitative PCR

To determine the persistence of MPXV nucleic acid on the different analyzed surfaces, DNA was extracted from all collected samples using the DNeasy Blood and Tissue kit (Qiagen) according to the manufacturer's protocol. After the wash steps, columns were eluted with 50 μL buffer AVE and stored at −80 °C until use.

Eluates were run on the CFX96 Touch Real-Time PCR Detection System (Bio-Rad Laboratories, Inc.) with cycling conditions of 60 °C for 1 minute, 95 °C for 2 minutes, followed by 40 amplification cycles of 95 °C for 10 seconds and 56 °C for 40 seconds. A total 20-μL reaction mixture contained Luna Probe One step reaction mix (No ROX) and Luna WarmStart RT Enzyme Mix (New England Biolabs Inc.), 0.025 μL of UDG Enzyme mix, 10 μM of F3L forward (5′-CTC ATT GAT TTT TCG CGG GAT A-3′), 10 μM of F3L reverse (5′-GAC GAT ACT CCT CCT CGT TGG T-3′), 10 μM of F3L MPXV probe (5′- FAM-CAT CAG AAT CTG TAG GCC GT-BHQ1-3′), and 5 μL of template DNA from nucleic acid extractions. In each PCR run, a negative control without template DNA and positive control containing MPXV DNA extracted from a known MPXV-positive sample were included. A Ct threshold of ≥36 for defining clearance of viral DNA based on prior reports [20].

## RESULTS

Temperature is a major factor affecting the stability and persistence of poxviruses [4, 21], and recent studies have determined that the viable MPXV persists longer in cooler climates, whereas virus concentration drops significantly after a few days at warmer temperatures [6, 8]. Therefore, the stability of viable MPXV on different surfaces encountered in a clinical setting was evaluated at both 22 °C, representing controlled ambient RT, and at 4 °C, representing refrigerated conditions. For a maximum of 21 days following inoculation, infectious virus and MPXV nucleic acid were quantified using gauze, paper, and scrubs as the porous materials tested, and plastic, IV tubing, steel, outer surface of N95, and gloves as nonporous materials.

**Figure 1.**
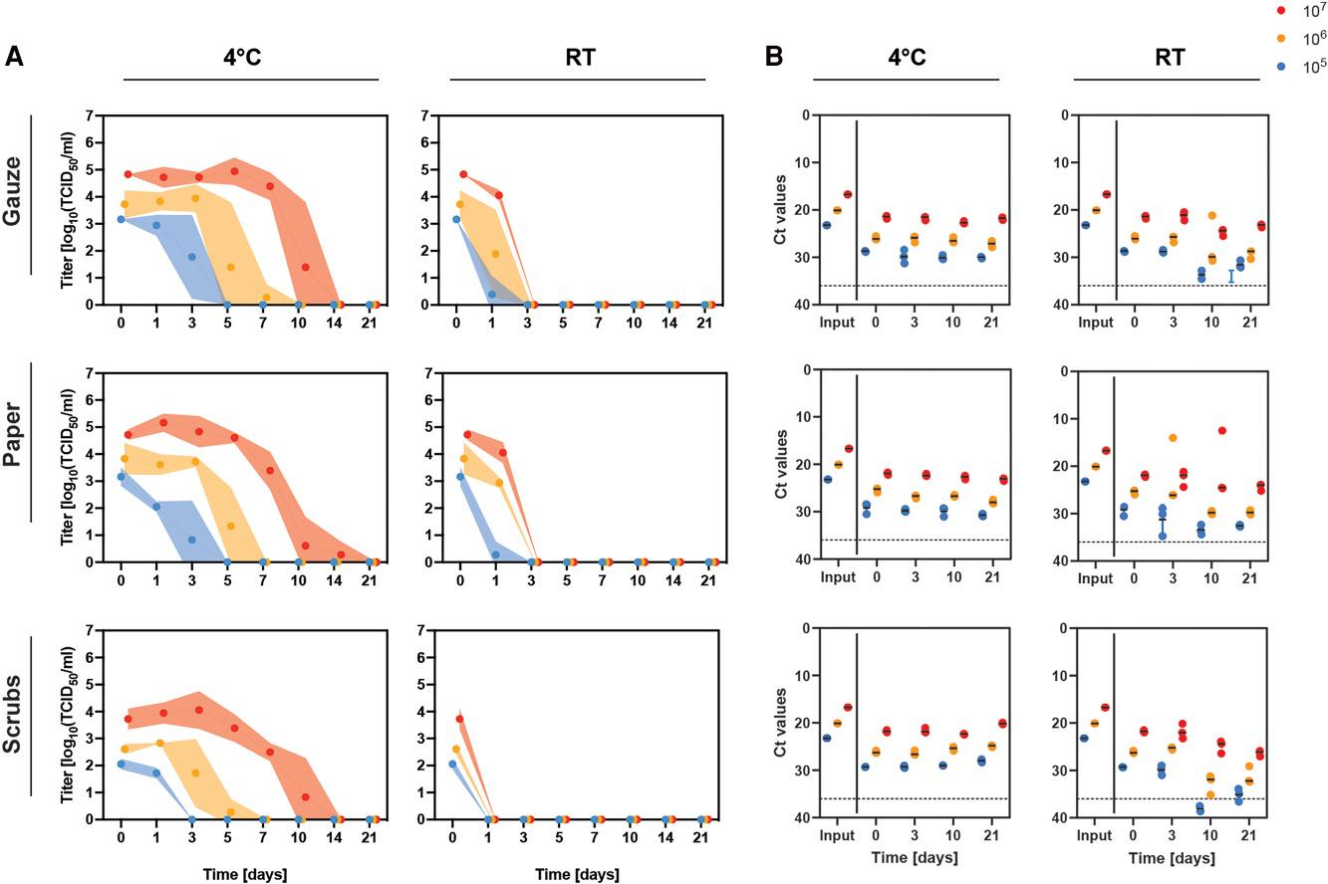
Stability of infectious monkeypox virus (MPXV) on porous surfaces at room temperature and 4 °C. Inocula of 50 μL containing 10^5^, 10^6^, or 10^7^ TCID_50_ of MPXV were evenly applied to the circular pieces of porous materials on 12-well plates in triplicate and incubated at room temperature (RT, 22 °C) or 4°C. *A*, Infectious MPXV titers and (*B*) MPXV DNA were measured up to 21 d after inoculation. *A*, The mean and standard deviation and area between error bands shaded is shown. *B*, The dashed horizontal line indicates a Ct value of 36, representing clearance of MPXV DNA.

On the porous materials, MPXV was less stable at RT, with infectious virus detected only at 1 dpi from gauze and paper, and no infectious MPXV detected from scrubs after inoculation (Figure 1*A*). MPXV viability decayed more slowly at 4 °C compared to RT, with infectious virus recovered up to 10 dpi on gauze and scrubs, and up to 14 dpi on paper from the 10^7^ TCID_50_ dose. There was a more rapid decrease of viable MPXV with the 10^6^ inoculum, with detection lasting up to 5 days on paper and scrubs and up to 7 days on gauze. For the lowest inoculum (10^5^ TCID_50_), viable MPXV was detected up to 3 days on gauze and paper, and up to 1 day on scrubs (Figure 1*A*).

Additionally, MPXV DNA was extracted from the eluted virus at 0, 3, 10, and 21 dpi. F3L gene Ct values gradually increased over time in samples incubated at RT. In contrast, Ct values remained constant on all tested porous surfaces at 4 °C, with minimal changes (<2 Ct increase) over time within each of the inoculum doses (Figure 1*B*). Ct values for all materials generally stayed below the limit of detection; however, after 10 days at RT, MPXV DNA was detected above the Ct threshold of ≥36 on scrubs from the 10^5^ TCID_50_ inoculum (Figure 1*B*). Overall, while viral loads declined at a more rapid rate at RT compared to 4 °C by 21 dpi, Ct values were similar at 1 and 3 dpi despite a marked decrease in infectious virus.

**Figure 2.**
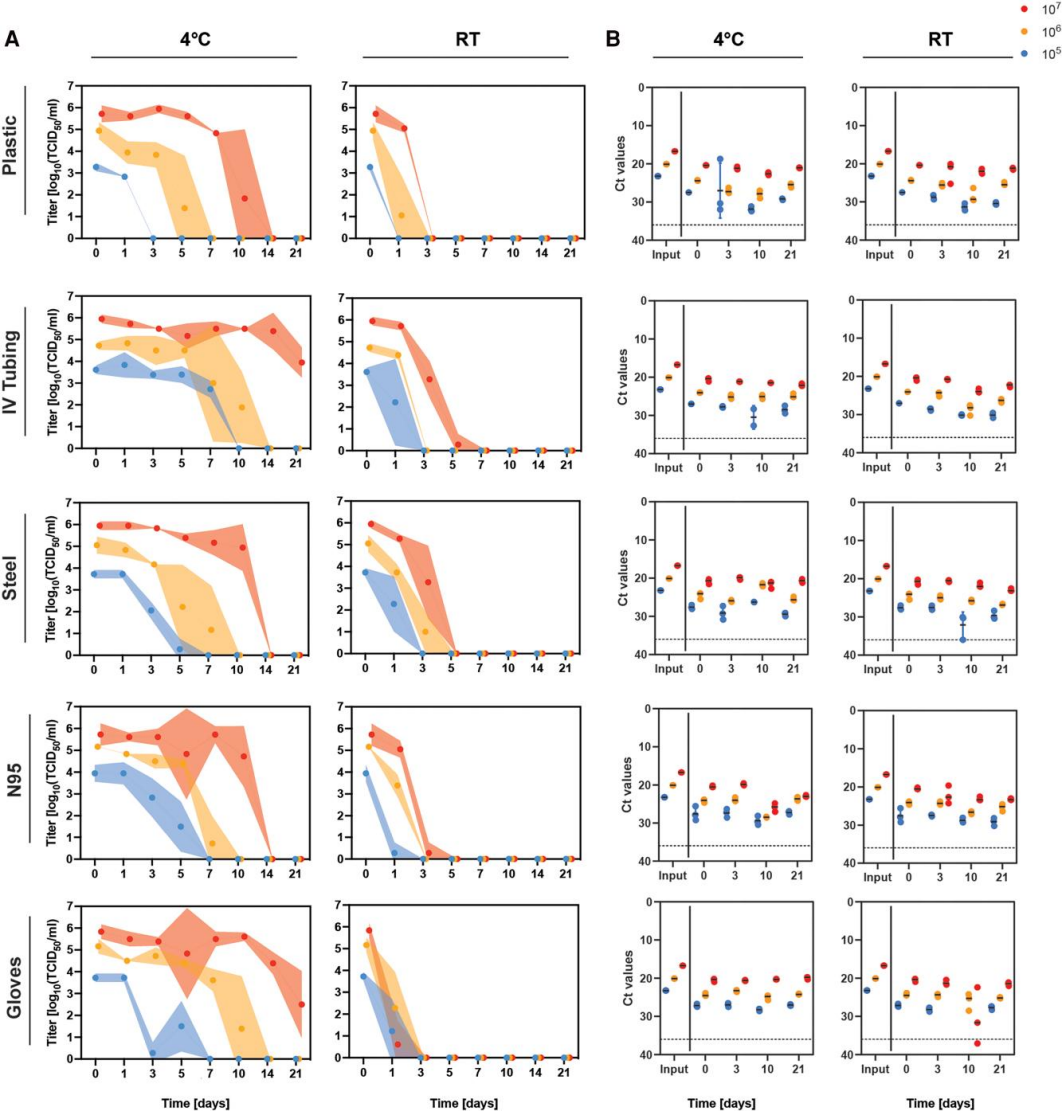
Stability of infectious monkeypox virus (MPXV) on nonporous surfaces at room temperature and 4 °C. Inocula of 50 μL containing 10^5^, 10^6^, or 10^7^ TCID_50_ of MPXV were evenly applied to the circular pieces of porous materials on 12-well plates in triplicate and incubated at room temperature (RT, 22 °C) or 4°C. *A*, Infectious MPXV titers and (*B*) MPXV DNA were measured up to 21 d after inoculation. *A*, The mean and standard deviation and area between error bands shaded is shown. *B*, The dashed horizontal line indicates a Ct value of 36, representing clearance of MPXV DNA.

On the nonporous materials, viable virus persisted the longest at 4 °C on intravenous tubing and gloves, with detection at 21 dpi, followed by plastic, steel, and the N95 mask, with detection up to 10 dpi, at the highest inoculum dose (10^7^ TCID_50_). Similarly, viable virus persisted the longest on intravenous tubing and gloves with detection up 10 dpi, up to 7 dpi for steel and N95 masks, and 5 dpi on plastic surfaces at 4 °C from the 10^6^ TCID_50_ inoculums. For the 10^5^ TCID_50_ inoculum, MPXV viability lasted for 7 days on intravenous tubing, 5 days for steel, N95s, and gloves, and only up to 1 dpi for plastic (Figure 2*A*). In contrast, MPXV titers rapidly declined on all surfaces after 1 dpi at RT. MPXV was most stable on IV tubing and steel with detection at 3 dpi with 10^7^ TCID_50_ but became rapidly undetectable after 1 dpi for all other surfaces tested (Figure 2*A*). As observed with the porous surfaces tested, Ct values remained comparable between 0 and 3 dpi and had minimally increased (<2 Ct) by 21 dpi despite a rapid decline in infectious virus particles (Figure 2*B*).

In summary, surface stability of MPXV was highly dependent on material and temperature. Generally, more rapid rates of decline and lower titers were recovered from surfaces incubated at RT compared to 4 °C. For porous surfaces, MPXV titers remained relatively constant for all materials tested for up to 5 days at 4 °C, but quickly declined after 1 dpi at RT. For nonporous surfaces, MPXV titers remained relatively constant for all materials tested for at least 7 days at 4 °C, but similarly declined after 1 dpi at RT. Altogether, the data suggests that MPXV is more stable at 4 °C compared to RT, and that Ct values are a poor predictor of infectious virus being present on a contaminated surface.

## DISCUSSION

Poxviruses are notoriously stable in the environment, which is influenced by several factors, including the viral burden, type of contaminated material, and environmental conditions [4, 5]. While previous studies have examined MPXV persistence on surfaces, most have focused on either wastewater or have been limited in surface types. This study provides insights into the environmental stability of MPXV on several porous and nonporous surfaces commonly found in clinical settings and representing many disposable materials that may be discarded as waste, providing a more comprehensive understanding of fomite-mediated transmission risks, including potential exposure of other mammalian species through waste and the establishment of novel reservoirs. Understanding the persistence of MPXV on these materials is essential for informing infection prevention and decontamination measures, particularly in healthcare settings, where the management and handling of materials contaminated by MPXV require stringent precautions to mitigate the risk of transmission. Fomites could pose a significant risk if they harbor sufficient viable virus where secondary infection can occur through various exposure routes, such as contact with mucous membranes or skin, especially if preexisting immunity is absent. The exact infectious dose of MPXV and lesion viral loads in humans are not well-established and inferred based on animal studies and related orthopox viruses (such as variola and VACV). In experimental infections of cynomolgus macaques with aerosolized MPXV, doses as low as 200 plaque-forming units (pfu) (∼290 TCID_50_, 0.69 pfu = 1 TCID_50_), which is similar to the infectious dose of variola virus (10–100 pfu or ∼15–150 TCID_50_) [22, 23]. Typically, vaccine doses of 2.5 × 10^5^ pfu (3.5 × 10^5^ TCID_50_) of replicating wild-type VACV have been given to induce immunity, although dilutions induce comparable responses [24]. In addition, VACV viral titers from lesion exudates in natural infections can exceed 10^7^–10^9^ TCID_50_/mL, and it is hypothesized that similar levels are shed in mpox lesions [25–27].

Here, we show that the stability of MPXV is highly dependent on both the material properties, such as porosity, and ambient temperature. Specifically, MPXV was found to be more stable at lower temperatures (4 °C) across all tested materials, with infectious virus detectable for significantly longer periods compared to RT (22 °C), consistent with previous studies on VACV, which also exhibit increased stability at lower temperatures on stainless steel and plastic [4, 21, 28].

On porous surfaces, including gauze, paper bedding, and cotton scrubs, MPXV showed a rapid decline in viability at RT, with viable virus only detectable up to 1 dpi on most materials. Notably, scrubs demonstrated the lowest titers of viable virus at room temperature, which suggest a reduced risk, but existing potential for transmission through clothing. In contrast, at 4 °C, MPXV persisted for up to 10–14 days depending on the material and initial viral load. These results indicate that porous materials may pose a lower risk for prolonged environmental transmission of MPXV in temperate conditions. The extended persistence of MPXV at lower temperatures on porous materials underscores the importance of maintaining strict decontamination protocols in cooler environments, such as sample storage areas within healthcare settings. Although gauze supported viable MPXV for only 1 day at room temperature, compared to up to 10 days at 4 °C, this may have implications for risk of secondary spread during the changing of bandages on lesion sites. Studies have shown that the use of gauze was associated with significantly higher rates of retrieval of VACV from the bandage up to 3 weeks after vaccination, and secondary spread from dressings has been reported [29, 30].

Nonporous surfaces representing disposable materials such as polypropylene plastic, intravenous tubing, stainless steel, the outer surface of N95 masks, and nitrile gloves, exhibited greater stability of MPXV, particularly at 4 °C. Viable virus was recoverable for up to 21 days on intravenous tubing and gloves, and up to 10 days on plastic, steel, and N95 masks at the highest inoculum dose (10⁷ TCID_50_). Prolonged viability of MPXV on nonporous surfaces could serve as reservoirs for transmission if not adequately disinfected, particularly in cooler environments. Meister et al. found that MPXV survived on stainless steel disks up to 30 days when stored at 4 °C compared to storage at RT, where MPXV declined rapidly after 5 days and was only detected up to 10 days [8]. Similarly, MPXV was less stable at higher temperatures, and more stable on stainless steel and polypropylene surfaces compared to cotton [9]. Another report shows that MPXV was more stable on smooth nonporous surfaces, such as glass, stainless steel, and plastic, than on porous and water absorbent surfaces, such as cotton fabric, cardboard, and wooden board [7]. The authors hypothesized that the high water-absorption capacity of these materials may have resulted in the inoculum droplet being adsorbed, which is consistent with our findings, as the proportion of infectious MPXV immediately after inoculating porous materials was lower than MPXV recovery from nonporous materials. Similar findings were observed in a VACV study, which found that the virus remained remarkably stable in the tested media (phosphate-buffered saline, double distilled water, and maintaining medium (2% FBS in DMEM) [28].

Although the risk of transmission in hospital settings outside of endemic areas is low, several incidents of healthcare providers contracting mpox have been reported in the current outbreak [13, 31–33]. Though in some cases, the primary modes of transmission were by needlestick injury, there were cases where it was suspected that contact with contaminated fomites was the route of transmission. Furthermore, evidence of replication-competent virus from air and floor samples collected during a bedding change has been found [34], as well as the gloves after contact with patient fabrics, indicating that cleaning and other procedures could cause contact with and reaerosolization of viable virus. Widespread contamination with MPXV DNA in isolation rooms of patients with symptomatic mpox has been shown [34, 35], as well as on surfaces touched by MPXV patients, including toilet seats, taps, and mobile phones [35]. These highlight the risk faced by healthcare workers and the importance of following proper personal protective equipment protocols, particularly during doffing, to prevent accidental exposure. Understanding the risk of transmission and probable exposure scenarios in healthcare settings is essential for Infection Prevention and Control measures, in addition to developing guidelines for post-exposure monitoring and prophylaxis. Furthermore, contaminated disposable materials may extend the risk of exposure to humans and other mammals outside facilities in the absence of adequate waste disposal.

This study highlights a discrepancy between MPXV nucleic acid detection via quantitative PCR (qPCR) and the presence of infectious virus. While prior reports suggest Ct values ≥36 indicate lower infectivity, we found no clear correlation, as MPXV DNA was detected at Ct <36 on most surfaces up to 21 dpi, regardless of viable virus. Ct values declined faster at RT than at 4 °C but remained stable at 4 °C across surfaces. Despite similar Ct values for porous and nonporous materials, prior research suggests higher viral DNA on non-porous surfaces [7]. These findings underscore qPCR's limitations in assessing infectious virus levels and the need for culture-based assays to evaluate transmission risk.

Our study has several limitations. First, these studies were performed using viral stocks in controlled conditions, and therefore the role of other biological materials (eg, blood, semen, saliva) in preserving the stability of infectious virus requires further investigation. Second, our experiments examined persistence of an MPXV clade II, and similar studies should be performed using MPXV clade I to ensure that there are not significant differences. In addition, MPXV viability in in vitro assays using VeroE6 cells may not always equate to infectivity in humans.

Considering the continuing worldwide mpox outbreak, these results have significant implications for public health strategies. The higher stability of MPXV on nonporous surfaces at low temperatures suggests that healthcare facilities and other environments where people with mpox are present should maintain strict cleaning and disinfection procedures, especially in areas where temperatures are regulated at lower levels. Additionally, the findings support the need for caution in the interpretation of qPCR results from environmental samples, as they may not accurately reflect the presence of infectious virus. Our study underscores the importance of environmental factors in the stability and potential transmission of MPXV and provides valuable data for guiding infection control and waste management measures in clinical and public health settings, highlighting the need for targeted decontamination efforts based on specific surface types and environmental conditions. Further research is warranted to explore the mechanisms underlying the differential stability of MPXV on various materials and to develop additional methods for assessing the risk of fomite-mediated transmission.

## Notes

***Acknowledgments.*** The authors are grateful to the Toronto High Containment Facility staff, Jessica Lam, Isobel Reckers, and Angel Ly for their technical help.

***Author contributions.*** S. M. and R. A. K. were involved in funding acquisition and project administration, study design, data interpretation, and supervision. A. B. was involved in study design, data collection, analysis, interpretation, and figure preparation. A. B. and J. R. H. were involved in writing the original draft and data collection. S. M., R. K., A. B., and J. R. H. were involved in manuscript revisions. E. C. and W. Y. were involved in data generation and analysis. K. N. was involved in data analysis.

***Data availability.*** Data will be made available from the corresponding author upon reasonable request.

***Financial support.*** This study was funded by the Canadian Institutes of Health Research (CIHR) Team Grant: Mpox (monkeypox) and zoonotic threats, application #495129. AB was funded by a CIHR Fellowship. RK and JRH were supported by the CIHR Team Grant (#495087).

***Potential conflicts of interest**.* All authors: No reported conflicts.
